# The Past, Present, and Future of Biomarkers for the Early Diagnosis of Pancreatic Cancer

**DOI:** 10.3390/biomedicines12122840

**Published:** 2024-12-13

**Authors:** Federica Vitale, Lorenzo Zileri Dal Verme, Mattia Paratore, Marcantonio Negri, Enrico Celestino Nista, Maria Elena Ainora, Giorgio Esposto, Irene Mignini, Raffaele Borriello, Linda Galasso, Sergio Alfieri, Antonio Gasbarrini, Maria Assunta Zocco, Alberto Nicoletti

**Affiliations:** 1 CEMAD Centro Malattie dell’Apparato Digerente, Medicina Interna e Gastroenterologia, Dipartimento di Medicina e Chirurgia Traslazionale, Università Cattolica del Sacro Cuore, Fondazione Policlinico Universitario “A. Gemelli” IRCCS, 00168 Rome, Italy; federica.vitale@guest.policlinicogemelli.it (F.V.); lorenzo.zileridalverme@policlinicogemelli.it (L.Z.D.V.); mattia.paratore@guest.policlinicogemelli.it (M.P.); marcantonio.negri@policlinicogemelli.it (M.N.); enricocelestino.nista@policlinicogemelli.it (E.C.N.); mariaelena.ainora@policlinicogemelli.it (M.E.A.); giorgio.esposto@guest.policlinicogemelli.it (G.E.); irene.mignini@guest.policlinicogemelli.it (I.M.); raffaeleborr@gmail.com (R.B.); linda.galasso@guest.policlinicogemelli.it (L.G.); antonio.gasbarrini@unicatt.it (A.G.); alberto.nicoletti@policlinicogemelli.it (A.N.); 2 Centro Pancreas, Chirurgia Digestiva, Dipartimento di Medicina e Chirurgia Traslazionale, Università Cattolica del Sacro Cuore, Fondazione Policlinico Universitario “A. Gemelli” IRCCS, 00168 Rome, Italy; sergio.alfieri@unicatt.it

**Keywords:** pancreatic cancer, biomarkers, CA 19-9, liquid biopsy, omics sciences, personalized medicine

## Abstract

Pancreatic cancer is one of the most aggressive cancers with a very poor 5-year survival rate and reduced therapeutic options when diagnosed in an advanced stage. The dismal prognosis of pancreatic cancer has guided significant efforts to discover novel biomarkers in order to anticipate diagnosis, increasing the population of patients who can benefit from curative surgical treatment. CA 19-9 is the reference biomarker that supports the diagnosis and guides the response to treatments. However, it has significant limitations, a low specificity, and is inefficient as a screening tool. Several potential biomarkers have been discovered in the serum, urine, feces, and pancreatic juice of patients. However, most of this evidence needs further validation in larger cohorts. The advent of advanced omics sciences and liquid biopsy techniques has further enhanced this field of research. The aim of this review is to analyze the historical evolution of the research on novel biomarkers for the early diagnosis of pancreatic cancer, focusing on the current evidence for the most promising biomarkers from different body fluids and the novel trends in research, such as omics sciences and liquid biopsy, in order to favor the application of modern personalized medicine.

## 1. Introduction

Pancreatic cancer (PC) is a leading cause of cancer-related mortality worldwide, with an increased global burden in the last decades and a dreadful prognosis due to the lack of adequate tools for identifying populations at risk and early diagnostic methods, as well as the poor response to treatments [1]. Indeed, in about 85% of cases, the disease is already locally advanced or metastatic at the time of diagnosis and is thus not susceptible to surgical treatment, which is the only one that significantly improves survival and quality of life in combination with oncological therapy protocols. A complete resection (R0) with disease-free resection margins is only possible in 15–20% of patients presenting with resectable PC. However, even after an optimal surgical intervention, 80% of patients relapse within 2 years. For patients who are not eligible for surgery, the prognosis is definitely worse, as oncological therapy alone does not determine a significant increase in 5-year survival. Evidence regarding adjuvant chemotherapy with gemcitabine, FOLFIRINOX, and nab-paclitaxel has demonstrated only a modest improvement in overall survival and progression-free survival, with a high grade of toxicity, such as neutropenia, asthenia, neuropathy, and diarrhea. The lack of efficient treatments for the advanced stage of disease has always been an impetus for the research and validation of novel biomarkers for the early identification of pancreatic cancer in the last decades and nowadays [2].

Carbohydrate antigen 19-9 (CA19-9) is a validated diagnostic biomarker of PC, which is currently the reference standard in clinical practice in association with imaging techniques. However, it does not guarantee effective sensitivity and specificity, and it has many limitations in the early identification of disease [3]. For this reason, novel biomarkers that are implicated in the early stages of carcinogenesis and selectively expressed in a large percentage of tumors have been investigated through the application of different revolutionary techniques and analysis in different body fluids in recent years [4]. In particular, the fascinating idea of non-invasive diagnosis has focused efforts on the study of biomarkers from different body fluids. Indeed, the anatomic location of the gland and the high expertise required for invasive diagnosis of PC are other limitations for the early diagnosis of the disease [5].

Moreover, the recent advent of omics techniques is expected to open novel perspectives in the precise non-invasive diagnosis of PC, offering clinical implications for personalized therapy [6].

The aim of this review is to summarize the importance of biomarkers for the early diagnosis of PC, focusing on the history that led to the biomarkers that are currently used in clinical practice and discussing promising research paths in this field for the development of future biomarkers.

## 2. The Past

A biomarker is a biological indicator that correlates with a given disease or response to a given treatment. To be valid, a marker should be able to be measured precisely, reliably, and quickly, and it should have high prognostic or predictive value and be able to predict the presence of a disease or its evolution, in the case of a disease marker, or to give indications on the most suitable type of drug and the response, in the case of a marker of response to treatment [7].

Until the 1970s, the diagnosis of PC was made by exploratory surgery. The only supportive biomarker for the diagnosis of PC of the head of the pancreas was serum bilirubin, despite its value being significantly nonspecific [8].

### 2.1. Carcinoembryonic Antigen (CEA)

Carcinoembryonic antigen (CEA) is a fetal glycoprotein with a described role in surveillance and the prognosis of colorectal cancer and potential implications in other neoplasms, such as ovarian, cervical, lung, and breast cancers [9]. Due to its scarce production after birth, its diagnostic value was also investigated in PC. CEA was one of the first serum biomarkers that was associated with PC with encouraging supportive results, particularly in advanced disease [10].

However, since the 1980s, the receiver-operating characteristic (ROC) curves demonstrated that CA 19-9 is more discriminating than CEA, for any serum value. Hence, CEA determination was considered unsatisfactory compared to CA 19.9 [11]. Hence, its use for the diagnosis of PC was abandoned. More recently, the use of CEA in combination with CA 19.9 offered a slightly higher diagnostic accuracy, but the sensitivity was not better than CA 19.9 alone [12]. Endorsing these conclusions, the current European Society for Medical Oncology (ESMO) guidelines do not recommend the use of CEA in PC management [13].

CEA determination in cystic fluid still plays a role in the diagnosis of mucinous pancreatic cystic neoplasms [14].

### 2.2. Carbohydrate Antigen 125 (CA 125)

Carbohydrate antigen 125 (CA 125) is a high-molecular-weight mucin-like glycoprotein that has been associated with ovarian cancer, colorectal cancer, and cholangiocarcinoma [15,16,17]. Its levels are increased in about half of patients with PC, and its combination with CA 19.9 increases the sensitivity by 6% when compared with CA 19.9 alone [18]. Unlike CA 19.9, it has no correlation with serum bilirubin levels; thus, it is not influenced by jaundice [18]. However, since CA 125 is also produced by the serosal epithelia, serum CA 125 levels are increased when serosal fluid is present, which is not uncommon in patients with PC [19].

Small studies reported a potential role of CA 125 in predicting PC resectability and predicting metastasis-associated disease burden and recurrence after resection [20,21]. However, this evidence is still far from being applied in clinical practice. Interestingly, patients with both neoantigens in the tumor antigen CA 125 and an abundance of CD8+ T-cell infiltrates had longer survival. Moreover, the loss of CA 125 neoantigenic clones was associated with tumor progression and metastasis [22].

### 2.3. Other Biomarkers

Carbohydrate antigen 50 (CA 50) is a ganglioside glycoprotein that is expressed on tumor cell surfaces. The CA50 epitope is assumed to be similar to the CA19-9 epitope (sialyl-Lewis a) and showed comparable diagnostic performance for PC [23]. However, in contrast to CA 19.9, it can be expressed by non-gastrointestinal cancers [24]. Due to the significant diagnostic overlap with CA 19.9, its use has declined since the 1990s.

In small populations, serum immunoreactive elastase 1 (IRE) was found in about 70% of patients with PC. However, it is also expressed in patients with pancreatitis [25]. Hence, it is not used in clinical practice.

## 3. The Present

### 3.1. Carbohydrate Antigen 19-9 (CA 19-9)

Since the 1980s, CA19-9, also called sialyl Lewis a (sLea) has been the current gold-standard biomarker for PC [26,27]. It is commonly used for PC diagnosis in symptomatic patients, assessment of prognosis, resectability, and monitoring of therapy because of its demonstrated correlation with tumor burden [28,29]. In 1979, Koprowski and colleagues derived it from a monoclonal antibody synthesized by a hybridoma obtained from mouse spleen inoculated with a human colorectal cancer cell line [30].

CA 19-9 is a high-molecular-weight glycolipid that expresses as its epitope a sialylated form of the Lewis a antigen (sialyl Lea) of the Lewis blood group system. It is the ligand for selectins E and P on endothelial cells, mediating the binding of tumor cells to endothelial cells that leads to systemic spread; thus, it is involved in the development of metastasis [31,32,33]. Its biosynthesis is strongly affected by β1,3-galactosyltransferases (B3GALTs), α(2,3) sialyl-transferases (S3TOs), and α(1,3/4) fucosyltransferase (FUT3): the activity and the location of these glycosylation enzymes and the amount of substrate can lead to an abnormal glycosylation that is closely related to PC development [34,35,36,37].

CA 19-9 is physiologically synthesized by fetal tissues, pancreatic and biliary ductal cells, and by gastric, colonic, endometrial, and salivary epithelia, so it is normally present in minimal concentrations in the blood (a normal cut-off of <37 U/mL is mostly used) [34]. In neoplasms, the alterations of some processes that regulate the production and passage into the circulation of mucins determine an increase in its serum levels. Moreover, it is implicated in the malignant evolution of PC because of its role in modifying proteins, affecting hematogenous metastasis by binding to selectin, mediating the immunological response, and facilitating angiogenesis [38,39,40].

During the last decades, conspicuous evidence has clarified its diagnostic and prognostic performance for PC. The sensitivity of CA19-9 for PC is reported to be in the range of 70% to 95% with a specificity between 72% and 90%. Moreover, several studies demonstrated a good potential for CA 19-9 in detecting early-stage PC with a median sensitivity of 76.1% (AUC 0.89) and a median specificity of 82%, with increased sensitivity up to 80.1% when considering all stages of PC [41,42].

In addition to diagnostic guidance, CA19-9 levels are also useful in the evaluation of tumor resectability in combination with clinical staging. In a study by Ferrone et al., preoperative CA19-9 levels correlated with the stage of the tumor and can predict survival in patients with resectable PC. The median preoperative CA19-9 was lower in patients without lymph node involvement (9 vs. 164 IU/mL, *p* = 0.06) and in patients with stage I-II compared to patients with stage III (41 vs. 162 IU/mL, *p* = 0.03). If the pre-intervention CA19-9 was less than 1000 IU/mL, there was a gain in survival of 16 months (*p* = 0.01) [43]. Another study by Berger et al. showed significantly poor survival for patients with postoperative CA19-9 higher than 180 (HR, 3.53; *p* < 0.0001) and for patients with CA19-9 higher than 90 (HR, 3.4; *p* < 0.0001) [44]. Additionally, high postoperative CA19-9 levels were shown to have a significant correlation with microscopically positive surgical margins and hepatic or peritoneal recurrence [45]. In another study, the resectability rate during surgery compared to imaging decreased as the preoperative CA 19-9 levels increased (CA19-9 levels, U/mL, <5, 73.7% resectability rate; 5–37, 79.7%; 37–100, 83.3%; 100–250, 82.2%; 250–500, 72.1%; 500–1000, 67.4%; 1000–2000, 61.1%; 2000–4000, 45.7%; and ≥4000, 38.3%) [28]. In a study by Kilic and colleagues, the average CA19-9 was 68.8 U/mL in the group of resectable patients (n = 18) and 622 U/mL in the unresectable group (n = 33) [46]. Since preoperative CA 19-9 >500 IU/mL was associated with a worse prognosis after surgery, despite the fact that no precise cut-off has been defined, an international consensus discouraged surgery in these cases [47]. This recommendation has been endorsed by the ESMO guidelines for the management of PC [13].

Furthermore, CA 19-9 demonstrated a good prognostic accuracy for the evaluation of the efficacy of neoadjuvant therapy. A decrease in the level of >50% seems to be associated with R0 (OR = 4.2; *p* = 0.05) after neoadjuvant therapy. Moreover, they could also predict histopathologic response and survival benefits, as described by Takahashi et al. [48,49].

Pancreatic cancer recurrence is strongly and promptly predicted by CA 19-9 elevation during follow-up. Reductions in CA19-9 values postoperatively are also an important predictive factor for the patient’s long-term prognosis compared to the levels at diagnosis [50].

From the perspective of monitoring therapy, postoperative CA19-9 levels were tested for their utility in determining the response to adjuvant chemotherapy for resected PC and in assessing the efficacy of systemic chemotherapy in advanced PC. Humphris et al. found that patients with postoperative CA19-9 levels ≤90 IU/mL had a good response to adjuvant chemotherapy (median 26.0 vs. 16.7 months, *p* = 0.011) after one month of administration, whereas those with postoperative CA19-9 levels >90 IU/mL had a poor response (16.2 vs. 9.0 months, *p* = 0.719) [51]. Conroy et al. evaluated the PC response to mFOLFIRINOX as adjuvant treatment and suggested that patients with postoperative CA19-9 levels ≤90 IU/mL benefited more from mFOLFIRINOX than from gemcitabine (HR 0.61, 95% CI, 0.48–0.77). Similarly, patients with any CA19-9 decline at week 8 of chemotherapy had a better prognosis than those without a decline in CA19-9 at week 8 (median 11.1 versus 8.0 months; *p* = 0.005), supporting the hypothesis that a CA19-9 decrease >15% was a reliable indicator of a more favorable outcome in patients treated for advanced PC who received chemotherapy. Moreover, it was an independent prognostic predictor for both overall survival (OS) and progression-free survival (PFS) (HR 1.92 and 2.15, *p* < 0.001, respectively) [52].

As previously mentioned, CA 19-9 is supposed to have a role in PC progression and metastasis. Based on this hypothesis, CA19-9 is an attractive therapeutic target for different antibodies and vaccines through the blockade of CA19-9/E-selectin-mediated systemic metastasis and the inhibition of CA19-9 biosynthesis. Sawada et al. identified the fully human monoclonal antibody 5B1 that specifically targets CA19-9 from circulating lymphocytes in subjects immunized with an sLea-KLH vaccine and showed an antitumor effect against CA19-9-positive cancer [53]. Similarly, cimetidine, a histamine type 2 receptor antagonist, was investigated as a potential suppressor of the metastasis of tumor cells by inhibiting the expression of E-selectin on endothelial cells and critical enzymes in CA 19-9 biosynthesis, such as FUT3. When downregulated, it can reduce the adhesion capacity of cancer cells and the colonization of the liver by tumor cells [54,55].

For all these reasons, CA 19-9 has represented the current and validated gold-standard biomarker for PC since its discovery. However, its US Food and Drug Administration approval was based on its performance in monitoring the response of PC to treatment, but its performance as a potential screening test for PC has been considered suboptimal. In the application of CA 19-9 as a diagnostic biomarker of PC, different limitations have been found, suggesting the need for further investigation to improve its performance. In particular, black tea consumption [56] or heavy tea consumption [57], liver damage [58], increased erythrocyte sedimentation rate [59], jaundice [60], bile duct obstruction and inflammation, pancreatitis [61], interstitial pulmonary disease [62], uncontrolled diabetes mellitus [63], various types of cystic tumors [64], collagen vascular diseases [65], endometriosis [66], hydro-nephrosis [67], colon diverticulitis [68], hypothyroidism [69], acute diarrhea, dyspepsia, gastric ulcer, and pulmonary fibrosis can elevate CA 19-9 values [70]. Moreover, aberrant levels of CA 19-9 have also been detected in other malignancies, such as colorectum, gastric, lung, breast, liver, and pancreatic neuroendocrine tumors, pointing out that CA 19-9 cannot be considered a tumor type-specific biomarker [71]. Additionally, the expression of the sialyl Lea antigen depends on the Lewis phenotype; therefore, approximately 5–10% of the population that is phenotypically negative for Lewis and does not have the Lewis fucosyltransferase enzyme is incapable of synthesizing CA 19-9, thus yielding false negative results for PC in Lewis-negative subjects [72].

Considering this evidence, new strategies for improving the CA19-9 diagnostic accuracy were performed: many studies have tried to combine CA19-9 with other biomarkers to improve the diagnostic performance of CA19-9, including other types of glycans, thrombospondin-1, thrombospondin-2, protein metabolite panels, and laminin-γC. To overcome the problem concerning the false negative cases related to Lewis-negative PC or Lewis-negative patients, CA 19-9 levels were combined with the Lewis genotype and other markers, such as duke pancreatic monoclonal antigen type 2 (DUPAN-2), sialyl Lewis x (sLex), CEA, CA 125 (CA 125), and the IgG/Gal ratio [73,74]. In Table 1 and Table 2, we summarized the merits and drawbacks of the use of CA 19-9 in clinical practice.

### 3.2. KRAS

According to the ESMO Clinical Practice Guidelines for pancreatic cancer, KRAS mutation is recognized as a genetic biomarker of disease because of its critical role in driving oncogenesis [13].

The KRAS gene encodes a member of the Ras family of small GTPases. It is mutated in 85% of PCs, critically impairing Ras GTPase activity, permanently activating the Ras protein, and maintaining the cellular processes of proliferation, transformation, invasion, and survival [75]. The evidence regarding the genetic analysis of clinical specimens suggests that the onset of this mutation is an early event in stage 1 pancreatic intraepithelial neoplasia (PanIN), confirming the role of the Ras signaling pathway as a key oncogenic driver of PC development [76].

Different biological samples, including fresh and fixed tumor tissue or biopsy samples, fine-needle aspiration materials and cytological samples, total blood, and plasma, can be used to detect KRAS mutation [77]. Even though multigene NGS is not currently recommended in patients with advanced PC in clinical practice, experts proposed its application in the context of molecular screening programs by clinical research centers as a tool to screen patients eligible for clinical trials, to accelerate drug development, and to increase the sensitivity, negative predictive value, and accuracy of cytopathology in the diagnosis of PC and differential diagnosis with chronic pancreatitis through the potential combination of the KRAS mutation assay with endoscopic ultrasonography-guided cytopathology [78].

In this context, the identification and characterization of the molecular profiles of KRAS-wild type metastatic PC with next-generation DNA/RNA sequencing, microsatellite instability (MSI), and mismatch repair status determination seem to be crucial to expand therapeutic options and offer targeted treatments in clinical practice. Real-world evidence suggests the frequent detection of different mutations within the DNA-damage repair (BRCA2, ATM, BAP1, RAD50, FANCE, PALB2), chromatin remodeling (ARID1A, PBRM1, ARID2, KMT2D, KMT2C, SMARCA4, SETD2), and cell-cycle control pathways (CDKN2A, CCND1, CCNE1) in KRAS-wild type PC, as well as gene fusions of BRAF (6.6%), FGFR2 (5.2%), ALK (2.6%), RET (1.3%), and NRG1 (1.3%) and amplification of FGF3 (3%), ERBB2 (2.2%), FGFR3 (1.8%), NTRK (1.8%), and MET (1.3%). Moreover, these targetable alterations provide a survival advantage for KRAS-wild type patients in overall cohorts and in patients treated with gemcitabine/nab-paclitaxel or 5-FU/oxaliplatin. Although in the case of the most common *KRAS* mutation, *KRAS G12D*, there have been several attempts to target this driver gene, specific inhibitors in monotherapy do not seem to be sufficient to control the disease because of a strong correlation between the presence of *RAS* mutations and resistance to immunotherapy, elucidating the rationale behind the promising results of combining KRAS inhibitors with immunotherapy [79].

## 4. The Future

The limitations in the performance of CA 19-9 in clinical practice and the lack of efficacy of treatments in the advanced stages of disease have encouraged research on novel biomarkers for PC.

From this perspective, through the application of omics techniques, nowadays, there are many studies in the literature that aim to find novel biomarkers for the early diagnosis, prediction of prognosis, choice of treatments, and monitoring of the response to therapies for PC, using different modalities for specimen collection for biomarker analysis [80].

**Figure 1:**
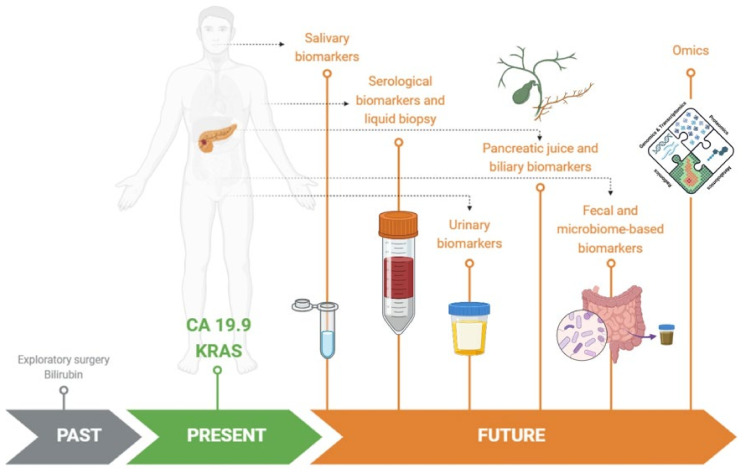
The past, present, and future of biomarkers of pancreatic cancer.

In Figure 1, we propose a graphical evolution of the research on biomarkers for the early diagnosis of pancreatic cancer.

In this section of the review, we discuss the main evidence from omics sciences and its application in the research of novel proteins isolated from different body fluids and identified as potential biomarkers of disease.

In Table 3, we also proposed an analysis of the limitations of different samples in the research on new biomarkers of disease.

### 4.1. Serologic Biomarkers

Among the different modalities for specimen collection, serum is the most commonly used medium because of the simplicity, low cost, and low risk of complications associated with sample collection. Despite the feasibility of analysis and research on biomarkers in serum samples through different methods of quantification and measurement of their plasma levels, some limitations depending on tumor marker dilution or obscuration by other serum proteins in the sample have to be considered [81].

In addition to the serum biomarkers already discussed in the previous sections, a large amount of glycolipids and proteins has been evaluated in the literature as potential biomarkers for the early diagnosis of PC. In this regard, osteonectin has been demonstrated to be involved in the mechanisms of invasion and metastasis of PC and to be able to detect PC at an early stage with a good sensitivity (84.6%) and specificity (87.5%), as well as secreted protein acidic and rich in cysteine (SPARC) [82,83].

Mucins are one of the most promising families of glycoproteins expressed by PC, with a possible diagnostic potential for early disease. They have a role in promoting metastasis, chemoresistance, and tumorigenesis [84,85]. In particular, MUC16 levels have a strong association with metastatic disease, whereas MUC5AC has great efficacy in differentiating resectable early-stage PC from healthy controls, with significant sensitivity for disease detection when combined with CA 19-9 and almost 100% specificity [86].

Likewise, apolipoprotein isoforms acting as lipid carriers and ligands for cell membrane receptors have been identified as promising serum biomarkers for PC diagnosis and prognosis [87]. APOE showed a sensitivity and specificity of 76.2% and 71.4%, respectively, for distinguishing patients with PC from healthy controls. Similarly, APOA2 was even better at detecting early-stage PC and identifying patients at high risk of pancreatic malignancy [88,89]. Higher APOC1 levels showed a correlation with poorer prognosis and independently predicted survival. Hence, its expression was considered a marker of aggressiveness in PC [90].

Insulin-like growth factor binding proteins 2 and 3 (IGFBP-2 and IGFBP-3) in a combined diagnostic panel had high discriminatory power in distinguishing intraductal papillary mucinous neoplasm (IPMN) and controls [91,92].

Protein expression already investigated in other neoplasms was also tested in patients with PC. Trefoil factors with ascertained roles in gastric cancer demonstrated significant elevation in PC compared to chronic pancreatitis patients and benign controls. Moreover, they showed a better accuracy in discriminating these populations when combined with CA 19-9 (AUC 0.93) [93,94]. Transthyretin, a carrier thyroxin, and triiodothyronine hormones that appeared to be elevated in endocrine tumors and decreased in epithelial ovarian carcinoma showed heterogenous levels of expression in PC patients [95]. PARK7/DJ-1, implicated in Parkinson’s disease and solid organ malignancies, presented with a good accuracy in identifying PC when compared to CA 19-9 (AUC 0.6647) [96].

Proteinase inhibitors, such as tissue factor pathway inhibitor, have been studied for their activity related to coagulation initiation. In multiple biomarker panels, their use in combination with tenascin C contributed to an improvement in the diagnostic performance of CA 19-9 [97]. On the contrary, the metalloproteinases involved in the regulation of cell proliferation and apoptosis, such as TIMP-1, showed lower sensitivity (47.1%), specificity (69.2%), and accuracy (AUC 0.64) than CA 19-9 in the detection of PC [98].

In the context of extracellular matrix (ECM), an upregulation of osteopontin (OPN) has been reported to have a good ability to distinguish PC from chronic pancreatitis and healthy controls, both alone and in combination with CA 19-9 [99,100,101]. In another study, laminin γ2 (LAMC2) levels was used in a diagnostic panel for PC in combination with CA 125 and CA 19-9. Its levels also showed an inverse correlation with OS in patients with PC [102]. In the analysis of glycoproteins that mediate cell-to-cell and cell-to-matrix interactions, CEA-related cell adhesion molecules (CEACAMs) 5 and 6, which are associated with the membrane through a glycosylphosphatidylinositol linkage, and 1, which is anchored to the cellular membrane by transmembrane domains, are expressed in a high percentage of PCs. However, there is scant evidence of their role in the detection of PC due to their overexpression in other solid organ malignancies [103,104,105]. ICAM-1 serum levels in PC have been evaluated in different studies as well, but its inability to distinguish between early- and late-stage PC limits its implementation as a screening and diagnostic biomarker [106]. In another study, the accuracy of thrombospondin-2 (THBS2) and thrombospondin-1 (THBS1) in the detection of PC was remarkable, with AUC values of 0.952 and 0.86, respectively. However, THBS2 showed no difference in expression between PC and distal cholangiocarcinoma, highlighting a potential diagnostic dilemma depending on a lack of specificity [107,108]. Similarly, despite the high levels of serum trypsinogen-2 in patients with PC and the high sensitivity (100%) of heat shock protein 27 (HSP27), serum amyloid A (96.5%), and M2-pyruvate kinase (85%) in the detection of PC, unfortunately, the elevation of their levels in chronic pancreatitis and benign obstructive disease highlighted a significant lack of specificity of these markers in the most common differential diagnosis in clinical practice [109,110,111,112].

Considering their implications in cell proliferation, pancreatic morphogenesis, epithelial mesenchymal transition, angiogenesis, and distant metastasis, growth factors represent promising diagnostic biomarkers of disease.

Zhao et al. observed higher levels of transforming growth factor-beta (TGF-β) in the sera of PC patients compared to benign controls and a significant correlation with poorer prognosis and reduced overall survival. Conversely, Yako et al. found heterogenous TGF-β levels, thus contradicting its possible use as a reliable biomarker [113,114]. Similarly, fibroblast growth factor 10/keratinocyte growth factor-2 (FGF-10/KGF-2) exhibited significant levels in the sera of patients with PC compared to controls. Vascular endothelial growth factor-A (VEGF-A) has been reported to be a predictor of distant metastasis and poor prognosis in PC [115,116]. Limited data are available about platelet-derived growth factor (PDGF) and tumor-specific growth factor. They seem to be associated with diagnostic superiority in the discrimination of PC when combined with CA 19-9, CA 242, and other interleukins [117,118].

In the study of the microenvironment, many cytokines and chemokines may play a key role as potential biomarkers of early disease. The serum expression of macrophage inhibitory cytokine-1/growth differentiation factor-15 was discovered to have comparable diagnostic accuracy to CA 19-9, with a sensitivity of 80%, specificity of 88%, and diagnostic odds ratio (DOR) of 24.57, with a moderately superior AUC (0.8945) in diagnosing PC and a moderate predictive capacity to identify high-risk patients for developing cancer, according to familial and genetic factors [119]. In the protein kinase pathways, CXCL11/interferon inducible T cell alpha chemokine overexpression in PC has been demonstrated to predict treatment response to gemcitabine and erlotinib [120]. Unfortunately, different studies highlighted the extreme variability and a lack of diagnostic ability of TNF-α and ILs for PC compared to biliary tract neoplasm and benign disease, and there is limited published literature concerning the elevated levels of oncostatin M (OSM), CXC motif ligand 8 (CXCL8/IL-8), stem cell factor (SCF), and macrophage colony-stimulating factor in PC [121,122,123].

Minimal evidence is found in the literature about other potential protein biomarkers. The levels of C4b-binding protein a-chain, implicated in B cell proliferation and CD40 activation to reverse immune suppression and stimulate anti-tumor T cell responses; colfilin-1, implicated in chemotaxis, cell migration, and tumor cell invasion; and soluble gC1qR (sgC1qR), implicated in inflammation and malignancy, have been reported in a single study to be significantly elevated in PC compared to chronic pancreatitis or healthy controls [124,125,126]. Leucine-rich a2-glycoprotein-1 (LRG1), soluble CD40 ligand (sCD40L), and aminopeptidase N were tested in a small study as early diagnostic biomarkers; however, larger sample sizes and a validation cohort are needed to confirm their diagnostic efficacy [127,128,129].

From the perspective of precision-targeted metabolomics, creatine, inosine, beta-sitosterol, sphinganine, glycocholic acid, and succinic acid were identified to improve the diagnosis and detection of disease progression in PC. Through the combination in a biomarker panel of 10 different blood metabolites, great accuracy in distinguishing PC patients from healthy controls, diabetic patients, and colorectal cancer patients (AUC values of 0.997, 0.992, and 0.653, respectively) was shown [130,131]. In Table 4 and Table 5, we summarized the most promising serologic biomarkers with their advantages and disadvantages for clinical applications.

### 4.2. Urinary Biomarkers

Urine is another easy-to-collect medium to identify potential specific biomarkers of PC. However, only a small amount of evidence regarding the urinary proteome is available in the literature. A combined biomarker panel composed of lymphatic vessel endothelial 1 (LYVE-1), hyaluronan receptor 1, REG1A, and thyroid transcription factor 1 was tested by Radon et al. and found to detect PC with AUC values of 0.89 and 0.92 in the training and validation datasets, respectively [132]. By replacing REG1A with REG1B in this panel, a higher accuracy (97%) for early PC detection was reached with a deep learning model.

Schneider et al. evaluated the urinary levels of TIMP-1, LYVE-1, and prostaglandin E Metabolite (PGEM) through the application of ELISA methods to distinguish patients with PC from IPMN and healthy controls: the median urinary TIMP-1 levels were significantly lower in healthy controls (n = 9; 0.32 ng/mg creatinine) compared to PC (n = 13; 1.95). However, they were not significantly different between low/moderate-grade (n = 20; 0.71) and high-grade/invasive IPMN (n = 20; 1.12). No significant difference in the urinary expression of LYVE-1 and PGEM was found between the groups [133]. The combination of four polyamines (acetylputrescine, diacetylspermidine, N8-acetylspermidine, and diacetylputrescine) distinguished pancreatic cancer and premalignant lesions of the pancreas from controls (sensitivity = 94%, specificity = 68%, and AUC = 0.88); the combination of diacetylspermidine, N8-acetylspermidine, and diacetylspermine distinguished acute pancreatitis from controls (sensitivity = 94%, specificity = 92%, and AUC = 0.98); the combination of acetylputrescine, diacetylspermidine, and diacetylputrescine distinguished chronic pancreatitis from controls (sensitivity = 98%, specificity = 71%, and AUC = 0.93) [134]. Interestingly, the electrolyte analysis of urine calcium and magnesium may also add some information for the identification of PC. Indeed, significantly lower levels were found in PC compared with healthy controls. These biomarkers identified metal dyshomeostasis with a sensitivity of 99.5% [135]. Hence, urinary biomarkers, combined with serum biomarkers, may enhance the diagnostic accuracy for PC [136,137]. The main results regarding this evidence are reported in Table 6.

### 4.3. Salivary Biomarkers

Saliva is emerging as an area interest in the field of biomarker detection as it provides a non-invasive means through which potential diagnostic biomarkers can be sampled. It has already found applications in other tumors, such as oral, breast, lung, ovarian, and esophageal cancer [138]. As for PC, Sugimoto et al. analyzed the saliva of 215 patients with different neoplasms and identified 48 metabolites as candidate biomarkers to discriminate PC with an AUC of 0.993 [139]. However, research on proteomic biomarkers in salivary fluid has shown some limitations because of the large amounts of salivary amylase, albumin, and immune-globulin contained in the saliva that extremely reduce the sensitivity in PC diagnosis [140]. The main results for this evidence are also reported in Table 6.

### 4.4. Pancreatic Juice and Biliary Biomarkers

An emerging and attractive modality of specimen collection is represented by pancreatic juice, which can be obtained through invasive procedures such as ERCP or endoscopy from the duodenum after secretin administration. Similarly, biliary tract fluid obtained from ERCP represents a potential source for biomarkers. Indeed, the presence and metabolic influence of PC may significantly alter their composition. However, the main limitations of the research on biomarkers in pancreatic juice and biliary tract fluid are the associated risk of induced pancreatitis, the risk of contamination of the sample with duodenal and gastric juice, and the reduced possibility of performing this technique in a large population of patients [141]. If the current literature about the assessment of biliary tract fluid biomarkers is scant, the expression of a large number of proteins, especially mucins, has been extensively evaluated in the pancreatic juice of patients with PC. Among them, Matsumoto et al. reported good results of KL-6 mucin expression in the pancreatic juice of patients with PC compared to inflammatory lesions and IPMN, despite a lack of specificity. In this regard, the heterogenous results obtained and the small cohort studies performed hinder their implementation as diagnostic biomarkers of disease [142]. The main results concerning this evidence are also reported in Table 6.

### 4.5. Pancreatic Cystic Fluid Biomarkers

In consideration of the risk of development of PC from pancreatic precancerous cystic lesions including IPMN and mucinous cystic neoplasms (MCNs), the analysis of pancreatic cystic fluid obtained from aspiration during EUS (EUS-FNA) has been proven to be a promising field of research for biomarker identification. However, this technique is limited by the amount of fluid aspirated and the size of the cyst, which can occasionally be insufficient [143]. The most important isolated proteins that have shown potential efficacy in discriminating premalignant and malignant lesions from benign ones are CEA, VEGF-A, and mucins. Among them, MUC4 may play a role in early carcinogenesis; thus, it is being used for the early detection of PC. Similarly, MUC1, MUC2, and MUC5AC were associated with PC when upregulated. On the other hand, MUC7 is significantly less specific as a diagnostic biomarker, since it was also found to be upregulated in IPMN and chronic pancreatitis [144,145,146,147]. The main results regarding this evidence are also reported in Table 6.

### 4.6. Fecal Biomarkers and Microbiota Analysis

The pancreas is a digestive organ, and thus, the occurrence of PC may influence the composition of stools and the fecal microbiota. Hence, among the different non-invasive methods of detection of novel biomarkers of disease, Haug et al. firstly focused on the rationale of using stool markers for the early detection of PC [148]. However, only a few studies focused on the use of this medium to identify biomarkers or metabolic signatures. miRNAs were detectable in fecal samples with high reproducibility. Lower concentrations of miR-216a, miR-196a, miR-143, and miR-155 were found in the feces of PC patients as compared to controls (*p* < 0.05). miR-181b and miR-210 were used to distinguish PC from healthy patients with ROC and AUC-ROC values of 0.745 and 0.772, respectively [149,150]. Scarce literature about fecal liquid biopsy and Kras mutations originating from the cells of pancreatic adenocarcinomas and from cells shed by abnormal pancreatic duct epithelium detected in the stool is now available [151]. Hence, further exploration of the fecal biomarkers’ role in screening assays for the early detection of pancreatic adenocarcinomas and precursors is needed.

In this scenario, fecal specimens are useful for the analysis of the microbiota, which has demonstrated significant perturbations in patients with PC and may have a possible role as a driver for carcinogenesis and progression of disease and thus a promising role as a diagnostic biomarker [152]. At present, there is scarce evidence about this field in the literature, but an abundance *Veillonella atypica*, *Fusobacterium nucleatum/hwasookii*, and *Alloscardovia omnicolens* and a depletion in *Romboutsia timonensis*, *Faecalibacterium prausnitzii*, *Bacteroides coprocola*, and *Bifidobacterium bifidum* were observed in the gut microbiota of PC patients as compared to healthy controls with an AUC of 0.84 in the distinction of the two groups [153]. Other interesting discoveries concern the microbiota-derived molecules’ influence on the efficacy of chemotherapy through the analysis of microbiota-derived tryptophan metabolite indole-3-acetic acid (3-IAA), which seems to be increased in patients who are responders to chemotherapy [154]. Hence, the study of the microbiota needs to be further explored because of its possible key role in helping to individualize PC care in the future. The main results of this evidence are also reported in Table 6.

**Table 6:** Urinary, salivary, pancreatic juice, pancreatic cystic fluid, fecal, and gut microbiota biomarkers.

Author	Biomarker	Sample	Results
**Radon et al. (2015) [132]**	Lymphatic vessel endothelial 1 (LYVE-1), hyaluronan receptor 1, REG1A, and thyroid transcription factor 1	Urine	AUC values of 0.89 and 0.92 in the training and validation datasets, respectively, to detect PC
**Yip-Schneider et al. (2020) [133]**	TIMP-1, LYVE-1, and prostaglandin E Metabolite (PGEM)	Urine	No significant difference in TIMP-1 levels between low/moderate-grade and high-grade/invasive IPMN; no significant difference in the urinary expression of LYVE-1 and PGEM between PC, IPMN, and healthy controls
**Nissinen et al. (2019) [134]**	N8-acetylspermidine, acetylputrescine, diacetylputrescine, and diacetylspermidine	Urine	Good accuracy in distinguishing PC and pancreatic premalignant lesions from controls (sensitivity = 94%, specificity = 68%, and AUC = 0.88)
**Sugimoto et al. (2010) [139]**	48 different metabolites	Saliva	Discriminated PC with an AUC of 0.993
**Matsumoto et al. (1994) [142]**	KL-6 mucin	Pancreatic juice	Higher expression in PC compared to inflammatory lesions and IPMN, despite the lack of specificity and small cohort studies
**Etekpo et al. (2018) [144]**	MUC4, MUC1, MUC2, MUC5AC, and MUC7	Pancreatic cystic fluid	MUC4 has a role in early carcinogenesis and the early detection of PC; upregulation of MUC1, MUC2, and MUC5AC in PC; lower specificity of MUC7 because of its upregulation in IPMN and chronic pancreatitis
**Ren et al. (2012) [149]**	miR-216a, miR-196a, miR-143, miR-155, miR-181b, and miR-210	Feces	Lower levels of miR-216a, miR-196a, miR-143, and miR-155 in PC patients compared to controls (*p* < 0.05); good accuracy of miR-181b and miR-210 in distinguishing PC from healthy patients (ROC 0.745, AUC-ROC 0.772)
**Kartal et al. (2022) [153]**	Veillonella atypica, Fusobacterium nucleatum/hwasookii, and Alloscardovia omnicolens	Gut microbiota	Abundance in PC patients compared to healthy controls (AUC 0.84)
**Tintelnot et al. (2023) [154]**	Tryptophan metabolite indole-3-acetic acid (3-IAA)	Gut microbiota	Increased in PC patient responders to chemotherapy

### 4.7. Liquid Biopsy

The term “liquid biopsy” refers to a minimally invasive methodology to obtain tumor-derived information from body fluids [155]. In this field, the most intriguing topic is represented by extracellular vesicles (EVs), which are lipid bilayer-coated globular organoids produced by all cells and contain molecules expressed by the originating cell (proteins, nucleic acids, lipids). They show different characteristics according to size and biogenesis: exosomes (30 to 150 nm) originate from the endocytosis of the plasma membrane and are discharged by exocytosis, and microvesicles (50 to 1000 nm) are generated via plasma membrane budding [156,157]. The growing interest in EVs concerns their release into the extracellular environment to mediate intercellular communication, the possibility of their isolation and identification in every biological fluid, their influence on metabolism and genome expression, and their enhancement of pathogenic mechanisms such as inflammation and carcinogenesis [158,159,160]. Promising studies about this topic have highlighted the potential role of EVs in favoring the epithelial–mesenchymal transition (EMT) through mediators such as lncRNAs, promoting lymphangiogenesis, promoting the progression of other cancer cells through an EV-mediated communication network and the recruitment of pancreatic stellate cells (PSCs) for the development of metastasis, activating mast cells to release inflammatory mediators, and regulating the immune response in PC [161,162,163,164].

In this regard, the identification of surface proteins and the analysis of the proteomic profiles of EVs are remarkably significant. Melo et al. identified glypican-1 (GPC1) as a diagnostic, prognostic, and predictive marker for disease-specific survival in PC (hazard ratio (HR): 5.353, CI: 1.651–17.358, *p* = 0.005). Indeed, significantly higher levels of GPC1+ exosomes were observed in PC patients, both at early and late stages, compared with benign pancreatic disease and healthy controls (*p* < 0.0001), with absolute sensitivity (100%–95% CI: 98.1–100%) and specificity (100%–95% CI: 97.1–100%), a positive predictive value of 100% (95% CI: 98.1–100%), and a negative predictive value of 100% (95% CI: 86.8–100%) [165]. However, heterogenous results about the ability to distinguish PC from benign lesions using GPC1+ EVs were obtained by Frampton et al. and Lucien et al., highlighting that further studies are needed to validate GPC-1 as a diagnostic biomarker of PC [166,167]. In the same direction, interesting studies investigated other possible PC-specific biomarker candidates, showing high levels of EVs expressing CLDN4, EPCAM, CD151, LGALS3BP, HIST2H2BE, HIST2H2BF, Annexin A6, CD151, and Tspan8. These subpopulations of EVs exhibited correlations with tumor progression, angiogenesis, and the formation of the pre-metastatic niche [168,169]. In addition, the exploration of the glycomic profile of EVs through a lectin microarray system detected the serum expression of O-linked glycosylation on mucin-1 (MUC1) and O-glycan-binding lectins *Agaricus bisporus* agglutinin (ABA) and *Amaranthus caudatus* agglutinin (ACA) as promising tools for the early diagnosis of PC [170,171]. Interestingly, Zheng et al. firstly identified multiple carcinoembryonic antigen-related cell adhesion molecules (CEACAMs), extracellular matrix (ECM) proteins (tenascin C, MMP7, LAMB3, LAMC2), and mucins (MUC1, MUC4, MUC5AC, MUC6, MUC16) in exosomes isolated from specimens of pancreatic juice [172].

In addition to the interest in proteomics, EV-derived nucleic acids showed a remarkable potential as reliable biomarkers for the early diagnosis and prognosis of PC due to the prolonged half-life of microRNAs (miRNAs, miRs) when encapsulated into EVs. In particular, miR-192-5p has been demonstrated to distinguish PC patients from healthy controls in exosomes (AUC = 0.83, *p* = 0.0004) with a diagnostic accuracy comparable to CA 19-9; unfortunately, it was not able to distinguish patients with PC and chronic pancreatitis [173]. Reese et al. observed the overexpression of miR-200b and miR-200c in the serum exosomes of PC patients compared with healthy controls (*p* < 0.001; *p* = 0.024) and chronic pancreatitis (*p* = 0.005; *p* = 0.19). Their combined diagnostic accuracy along with CA 19-9 reached 97% (*p* < 0.0001) in the prediction of PC [174]. Similarly, great sensitivity (71.1%) and specificity (96.9%) were obtained from miR-200b expression in exosomes in the distinction of PC from healthy controls, with a correlation between miR-200, SIP1, and E-cadherin expression [175]. Moreover, exosomal miRNAs were also investigated for their role in differentiating early-stage PC from healthy controls and advanced-stage PC, with an improved diagnostic value (AUC = 0.791, *p* < 0.0001) observed with the combination of miR-21 and miR-10b (*p* < 0.05, early stage vs. healthy; *p* < 0.001, early stage vs. advanced stage); in their prognostic role, correlations were observed for higher levels of EV-derived miR-17-5p with metastasis and advanced-stage PC and miR-200c in total serum exosomes with shorter overall survival (*p* = 0.038) [176,177]. Furthermore, an interesting evaluation of the exosomal surface antigen panel PaCIC (CD44v6, Tspan8, EpCAM, MET, and CD104) and serum exosome miRNA markers (miR-1246, miR-4644, miR-3976, and miR-4306) confirmed that the combination of different potential diagnostic tools can increase the sensitivity (1.00, CI: 0.95–1) and specificity (0.80, CI: 0.67–0.90) in the differentiation of PC from chronic pancreatitis, healthy controls, and non-pancreatic malignancies [178].

More recently, novel diagnostic biomarkers for PC were isolated from urine samples, revealing that the elevation of the miR-3940-5p/miR-8069 ratio was specific for PC at a relatively early stage of disease and tended to be higher in urine exosomes than in the serum of patients with PC; the sensitivity (93.0%) and positive predictive value (100%) were improved when in combination with elevated CA 19-9 [179].

Other applications of liquid biopsy concern the isolation from different body fluids of circulating extracellular nucleic acids (cell-free DNA—cfDNA) and circulating tumor DNA (ctDNA) that can be assayed using next-generation sequencing (NGS). The performance of circulating tumor cells (CTCs) as an adjunctive biomarker at the time of disease presentation was firstly investigated by Kulemann et al., who suggested their possible role in characterizing the genetic alterations of PC because of the ability to capture, cytologically identify, and genetically analyze CTCs [180]. Then, Ankeny et al. used KRAS mutation analysis to compare CTCs with primary tumor tissue: promising results revealed CTCs as a good diagnostic tool for PC (sensitivity = 75.0%, specificity = 96.4%, area under the curve (AUROC) = 0.867, 95% CI = 0.798–0.935, and *p* < 0.001) with a cut-off of ≥3 CTCs in 4 mL of venous blood to discriminate between local/regional and metastatic disease (AUROC = 0.885; 95% CI = 0.800–0.969; and *p* < 0.001) [181]. The detection of folate receptor (FR)-positive CTCs through ligand-targeted polymerase chain reaction (LT-PCR) showed significantly higher levels of FR+ CTCs in malignant diseases than in benign pancreatic diseases (*p* < 0.01), with a better diagnostic efficiency, high sensitivity (97.8%), and high specificity (83.3%) in the case of the combination of FR+ CTCs with CA 19-9 [182].

In recent years, liquid biopsy has also focused on DNA extraction from the urine of PC patients, with the hypothesis based on cfDNA originating from the shedding of cells from the genitourinary tract or through the kidney and the glomerulus: analysis of KRAS mutations with droplet digital PCR showed heterogeneous results according to renal functions, with a detection rate of urine KRAS mutations increasing in the group with a worse creatinine clearance rate [183]. Further applications concern the assessment of the diagnostic utility of telomerase activity in pancreatic juice, which was revealed to be a reliable biomarker in PC: the detection of mRNA for human telomerase reverse transcriptase (hTERT) has been validated in different studies as a diagnostic biomarker for PC because of the significant role played by telomerase reactivation in the development of hepatobiliary and pancreatic tumors [184,185]. Additionally, the assessment of the diagnostic accuracy of mutant KRAS oncogene detection from pancreatic juice in PC revealed a wide range of variation in sensitivity (38%–89%) and specificity (13%–100%); the DNA methylation status of MUC1, MUC2, and MUC4 for the differential diagnosis of human pancreatic neoplasms had a sensitivity and specificity of 87% and 80% for PC; and methylated ppENK and p16 were found in the pancreatic juice of patients with PC in 90.9% and 18.2% of cases, respectively [186,187,188]. Finally, Levink et al. highlighted the role of 8q24 gain-of-function mutation in the oncogene Myc in pancreatic juice as a promising biomarker for the detection of PC with a sensitivity of 33% (95% CI 16–55%) and a specificity of 94% (95% CI 70–100%) [189].

The improvement in these techniques might allow for performing a risk assessment of cancer cells present in the circulation of patients before cancer detection, for making a prospective screening population, and for preventing the progression of pancreatic cystic lesions to PC. In this scenario, DNA-based biomarkers, including KRAS and GNAS noted to be elevated in mucin-producing cysts, and supervised machine learning techniques might represent an innovative tool to develop a comprehensive test, CompCyst, based on selected clinical and imaging features combined with cyst fluid genetic and biochemical markers, to guide the management of pancreatic cysts. However, these promising applications are still far from being applicable in clinical practice and need further standardization and validation of the techniques [190,191,192]. Moreover, the ability to non-invasively sample tumor tissues might also help to tailor treatments from the perspective of personalized medicine. In Table 7, we synthesized the most promising exosome biomarkers in liquid biopsy, comparing the advantages and disadvantages for clinical applications for each of them.

### 4.8. Non-Coding RNAs

Non-coding RNAs include RNAs of different length that are not translated into proteins: long non-coding RNAs, with a length greater than 200 nucleotides, and micro-RNAs, composed of a sequence of 20–25 nucleotides produced from a precursor transcript by consecutive cleavage [193]. They are released into the circulation from tumor cells, even though the basis of this mechanism is still unclear, and they can possibly be isolated from serum and other body fluids, such as cerebrospinal fluid, breast milk, saliva, and urine [194,195]. Non-coding RNAs have been shown to have a role in mediating cell-to-cell interactions, particularly modulating the processes of transcription, chromatin modification, gene transcription, post-translational modification, the regulation of gene expression and intracellular signaling pathways, promoting or suppressing tumor growth, and modulating both intrinsic and acquired chemoresistance [196,197]. Hence, a growing interest in the study of non-coding genetic materials, particularly micro-RNA (miRNA, miR), as biomarkers of disease has developed in the last years. 

Wang et al. profiled the plasma levels of four different miRNAs (miR-21, miR-210, miR-155, and miR-196a) that are potentially implicated in the development of PC, observing a sensitivity of 64% and a specificity of 89% in the identification of early PC [198]. Abue et al. confirmed the role of miR-21 in distinguishing PC patients from healthy controls (*p* < 0.01) and demonstrated its expression at higher levels at the advanced stage of disease (*p* < 0.05), in metastasis to the lymph nodes and liver (*p* < 0.01), and the shorter survival (*p* < 0.01) in patients with PC [199]. Similarly, Guz et al. and Ho et al. in their studies further supported the previous evidence about the increased levels of miR-210-3p in the sera of patients with PC compared with chronic pancreatitis patients (*p* = 0.015) and the control group (*p* < 0.001). They also showed a positive correlation between miR210-3p levels and alkaline phosphatase (r = 0.605; *p* = 0.022) and γ-glutamyltranspeptidase (r = 0.529; *p* = 0.029) and considered miR210-3p as a novel marker of hypoxia [200,201].

Yan et al. analyzed the expression profiles of 2.555 serum miRNAs in 100 pancreatic cancer patients and 150 healthy controls. They identified 13 pancreatic cancer signature miRNAs (miR-125a-3p, miR-6893-5p, miR-125b-1-3p, miR-6075, miR-6836-3p, miR-1469, miR-6729-5p, miR-575, miR-204-3p, miR-6820-5p, miR-4294, miR-4476, and miR-4792) that may classify the PC patients and healthy controls and 432 miRNAs that may predict the resectability of PC [202].

Since such mediators are rapidly degraded in the bloodstream, extracellular vesicle (EV)-derived micro-RNAs are considered a more reliable biomarker of disease. Panels of different mRNAs (miR-21, -34a, -99a, -100, -125b, -148a, -155, -200a, -200b, -200c, and -1246) have been tested to evaluate their diagnostic role and accuracy in comparison with CA 19-9 levels and their prognostic role in PC, identifying a possible correlation with a poorer overall survival and a more rapid progression of disease [174,203,204].

The interest in lncRNAs has increased in the last 10 years, because of their binding to gene promotors, their action as a miRNA sponge, and their influence on the biological behavior of cancer, with heterogeneous results obtained from their evaluation as potential diagnostic markers of PC and the difficulty of validation caused by single cohort studies. The lncRNA Linc-pint showed lower expression in plasma samples of PC patients compared with healthy individuals and adjacent tissues, carcinoma of the ampulla of Vater (CAV), and cholangiocarcinoma (CCA) and was correlated with tumor recurrence and poor prognosis after pancreatectomy [205]. Guo et al. and Shuai et al. demonstrated significant upregulation of lncRNA SNHG15 in PC with a potential role in differentiating PC tissues from normal pancreatic tissues and PC patients from healthy controls and in predicting tumor differentiation (*p* = 0.000), lymph node metastasis (*p* = 0.001), tumor stage (*p* = 0.005), and shorter overall survival (*p* = 0.003) [206,207]. LINC01638 showed an inhibitor effect on the migration and invasion of PC cells by reducing TGFβ signaling, HULC through the Wnt/β-catenin signaling pathway, and ABHD11-AS1 and UFC-1 lncRNAs and thus might serve as a potential serum biomarker for the diagnosis and prognosis of PC [208,209,210,211]. At present, no evidence about the urinary or pancreatic juice isolation of lncRNAs is available in the literature. Xie et al. identified salivary HOTAIR and PVT1 as novel non-invasive biomarkers with significantly higher levels in PC compared to healthy groups [212].

Debernardi et al. tested the miRNAs in urine specimens of patients with PC for non-invasive, early detection of disease. They identified a significant overexpression of three miRNAs (miR-143, miR-223, and miR-30e) in patients with stage I cancer compared with healthy controls (*p* = 0.022, 0.035, and 0.04, respectively) and with stages II-IV PC (*p* = 0.025, 0.013, and 0.008, respectively). The AUC of the combination of miR-143 and miR-30e was 0.923 (95% CI 0.793–1.000), with a sensitivity of 83.3% (95% CI 50.0–100.0) and specificity of 96.2% (95% CI 88.5–100.0) [213]. Sadakari et al. proved the existence of miRNAs in pancreatic juice and compared their expression levels in patients with PC and chronic pancreatitis: miRNA-21 and miRNA-155 were significantly higher in PC than chronic pancreatitis (*p* < 0.001 and *p* = 0.008, respectively), with AUC values of 0.90 and 0.89 and accuracy values of 83% and 89%, respectively. No correlation was demonstrated with the preoperative cytological results of pancreatic juice [214]. Moreover, in the analysis of miRNA biomarkers in pancreatic juice, Wang et al. observed a significant difference in the profiles of expression of four circulating miRNAs (miR-205, miR-210, miR-492, and miR-1427) between PC and healthy controls, predicting PC with a specificity of 88% and sensitivity of 87%. Among them, higher levels of expression of miR-205 and miR-210 predicted lymph node metastasis, representing promising candidate biomarkers of poor prognosis in patients with PC [215]. Humeau et al. identified that miR-21, miR-23a, miR-23b, and miR-29c were significantly upregulated in the saliva of patients with unresectable PC compared to controls, showing sensitivities of 71.4%, 85.7%, 85,7%, and 57%, respectively, and excellent specificity (100%) [216]. Xie et al. analyzed salivary miR-3679-5p and miR-940 as good discriminatory biomarkers to detect resectable pancreatic cancer, with reasonable specificity and sensitivity [217].

MiRNAs were also tested in distinguishing between premalignant and malignant lesions. Matthaei et al. studied potential candidate miRNAs (miR-18a, miR-24, miR-30a-3p, miR-92a, miR-99b, miR-106b, miR-142-3p, miR-342-3p, miR-532-3p) that helped in identifying patients with high-grade IPMN and excluding non-mucinous cysts. Farrell et al. identified miR-21 as a candidate biomarker to distinguish between benign, premalignant, and malignant cysts, even if this evidence will require validation in a prospective setting to ultimately confirm its clinical usefulness [218,219]. In Table 8, we clarified the positive aspects presented by the most promising non-coding RNAs for clinical application.

### 4.9. Omics Sciences

The advent of advanced technology in the field of biomedical research has opened the way for advances in the comprehension of diseases for which traditional approaches have failed to find tools and treatment that are able to modify the course of disease [6]. Recently, the development of omics technologies, such as non-targeted metabolomics, genome sequencing, tandem mass spectrometry molecular networking, and high-throughput screening, have also altered the natural products discovery landscape and their role as important sources of new drug development and alternative de-replication approaches for efficient natural products discovery [220].

Omics sciences play a key role in the investigation of PC pathogenesis, the distinction of different subtypes of PC according to molecular genetic and transcriptomic profiling and metabolic changes, the identification of a new panel of biomarkers using proteomics analysis, and the application of revolutionizing methods of the computational analysis of imaging [221,222,223].

Proteomics has offered opportunities for the identification of altered protein expression through comparative analysis between normal and pathological tissues and in the evaluation of secreted proteins and serum protein profiles. Such information may contribute to the study of the tumor microenvironment; the distinction of inflammatory, precancerous, and malignant lesions; the application of immune cells and cytokine expression in the field of immunotherapy; and the assessment of tumor burden. Indeed, when specific proteins with a role in carcinogenesis are expressed, a shorter OS, shorter PFS, and chemotherapy resistance are frequent [224,225]. Considering a direct application of proteomics in the optimization of vaccine immunogens, enzymes for sustainable chemistry, and proteins with therapeutic potential, despite the fact that proteins can exhibit complex folds defying atomistic design calculations, computational protein design accuracy has been improved by the advent of deep learning-based ab initio structure predictors to mitigate the risk of misfolding and aggregation [226].

Similarly, a better comprehension of the tumor microenvironment, carcinogenesis, and biological behavior of PC is possible through the investigation of cancer cell metabolic reprogramming, specifically glucose, lipid, and amino acid metabolism in response to oncogenic alterations that involve lower amino acid and carbohydrate levels or the enrichment of glycolysis and serine pathway components and the abundance of different lipid metabolites. These findings seem to be useful in identifying different PC subtypes, distinguish cancerous and non-cancerous conditions and PC from other neoplasms, and in the understanding of disease aggressiveness [227,228,229]. In this regard, relevant patient-derived models, including patient-derived xenografts (PDXs), patient-derived organoids (PDOs), and patient-derived explants (PDEs), play a crucial role in the comprehension of the intricate intercellular communication among tumor cells and the mechanisms underlying tumor growth, drug responsiveness, and individual patient sensitivities to enable personalized medical approaches [230]. In the future, this knowledge may help to individualize treatments and avoid useless and harmful therapies in patients with predictable non-responsiveness.

Moreover, the genomic landscape of PC has been deeply analyzed to find key mutations, such as KRAS, loss of TP53, and the inactivation of CDKN2A and SMAD4; gene overexpression and recombination, including C-Myc, BRCA 1/2, and PALB2; and mismatch repair deficiency in driving disease development, chemoresistance, intra-tumor angiogenesis, epithelial-mesenchymal transition (EMT), tumoral invasion, and metastasis [79,231,232].

The study of oncogenes represents a promising tool for a personalized approach and a direct application of new genomic evidence in the treatment in clinical practice, as already demonstrated for KRAS inhibitors and targeted therapies with activity against ROS1 [233,234].

Furthermore, the advent of new computational models based on mathematical methods of the extraction of quantitative data from acquired CT or MRI images represents a promising tool for diagnosis, staging, choice of treatments, response to treatments, and prognosis of PC [235,236,237,238]. For example, radiomics features can find application in the prediction of the risk of lymph node metastasis to avoid unnecessary surgery and postoperative complications and in the identification of good candidates for immunotherapy targeting immune checkpoint inhibitors [239,240,241,242].

## 5. Conclusions

In conclusion, the wide evidence available regarding the research on novel reliable biomarkers for the early diagnosis of PC underlines how this field is particularly thriving in the literature, following the growing number of cases and deaths from this tumor, which is estimated to become the second leading cause of death by 2030. Further studies will be needed to expand the population samples considered; confirm the sensitivity, specificity, and accuracy; and evaluate the reliability of these biomarkers. The main aim is to validate new biomarkers of disease, isolated from different body fluids, in order to anticipate the timing of diagnosis of PC, increase the number of patients that can be potentially cured with surgery, and improve their 5-year survival, which is currently still very dismal. Furthermore, the possibility of the application of these biomarkers for predictive purposes of the response to antineoplastic treatments and complications following surgical interventions lays the foundation for personalized medicine in this field.

## Figures and Tables

**Table 1:** Merits of CA 19-9 in clinical practice.

Author	Results
**Goonetilleke et al. (2007) [41]** **Kim et al. (2017) [42]**	Good potential in detecting early-stage PC with a median sensitivity of 76.1% (AUC 0.89) and a median specificity of 82%; increased sensitivity up to 80.1% when considering all stages of PC
**Ferrone et al. (2006) [43]**	Correlation of preoperative levels with the stage of the tumor and prediction of survival in patients with resectable PC; median preoperative CA19-9 lower in patients without lymph node involvement (9 vs. 164 IU/mL, *p* = 0.06) and in patients with stage I-II compared to patients with stage III (41 vs. 162 IU/mL, *p* = 0.03)
**Berger et al. (2008) [44]**	Poor survival for patients with postoperative CA19-9 >180 (HR, 3.53; *p* < 0.0001) and CA19-9 >90 (HR, 3.4; *p* < 0.0001)
**Hata et al. (2012) [45]**	Correlation of high postoperative CA19-9 levels with microscopically positive surgical margins and hepatic or peritoneal recurrence
**Hartwig et al. (2013) [28]**	Correlation of increased preoperative CA 19-9 levels with decreased resectability rate during surgery (CA19-9 levels, U/mL, <5, 73.7% resectability rate; 5–37, 79.7%; 37–100, 83.3%; 100–250, 82.2%; 250–500, 72.1%; 500–1000, 67.4%; 1000–2000, 61.1%; 2000–4000, 45.7%; and ≥4000, 38.3%)
**Boone et al. (2014) [48]** **Takahashi et al. (2010) [49]**	Good prognostic accuracy for the evaluation of the efficacy of neoadjuvant therapy and prediction of histopathologic response and survival benefits; levels decreasing >50% associated with R0 (OR = 4.2; *p* = 0.05) after neoadjuvant therapy
**Humphris et al. (2012) [51]**	Correlation of postoperative CA19-9 levels ≤90 IU/mL with good response to adjuvant chemotherapy (median 26.0 vs. 16.7 months, *p* = 0.011) after one month of administration
**Conroy et al. (2018) [52]**	Correlation of postoperative CA19-9 levels ≤90 IU/mL with more benefit from mFOLFIRINOX than from gemcitabine (HR 0.61, 95% CI, 0.48–0.77); role of CA19-9 decrease >15% as a reliable indicator of a more favorable outcome in patients treated for advanced PC who received chemotherapy; role of CA 19-9 as an independent prognostic predictor for both overall survival (OS) and progression-free survival (PFS) (HR 1.92 and 2.15, *p* < 0.001, respectively)

**Table 2:** Drawbacks of CA 19-9 in clinical practice.

Author	Results
**Isaji et al. (2017) [47]**	No precise cut-off defined to discourage surgery, despite correlation of preoperative CA 19-9 >500 IU/mL with a worse prognosis after surgery
**Al-Janabi et al. (2017) [56]** **Howaizi et al. (2003) [57]**	Elevation of CA 19-9 values because of black tea consumption or heavy tea consumption
**Basso et al. (1990) [58]** **Goh et al. (2017) [60]** **Mujica et al. (2000) [61]**	Elevation of CA 19-9 values because of liver damage, jaundice, bile duct obstruction and inflammation, pancreatitis
**Kim et al. (2009) [59]** **Totani et al. (2005) [62]** **Cantagrel et al. (1994) [65]**	Elevation of CA 19-9 values because of increased erythrocyte sedimentation rate, collagen vascular disease, and interstitial pulmonary disease
**Huang et al. (2012) [63]** **Tekin et al. (2002) [69]**	Elevation of CA 19-9 values because of uncontrolled diabetes mellitus and hypothyroidism
**Jones et al. (2009) [64]** **Alencar et al. (2019) [71]**	Elevation of CA 19-9 values because of various types of cystic tumors and other malignancies, (colorectal, gastric, lung, breast, liver, and pancreatic neuroendocrine tumors)
**Harada et al. (2002) [66]** **Inayama et al. (2006) [67]** **Nakamura et al. (2002) [68]**	Elevation of CA 19-9 values because of endometriosis, hydro-nephrosis, and colon diverticulitis
**Ventrucci et al. (2009) [70]**	Elevation of CA 19-9 values because of acute diarrhea, dyspepsia, gastric ulcer, and pulmonary fibrosis
**Luo et al. (2018) [72]**	False negative results in Lewis-negative subjects because of the absence of fucosyltransferase enzyme and the incapability of synthesizing CA 19-9

**Table 3:** Limitations of the most used samples in the research on potential biomarkers.

Sample	Limitations
Serum	Tumor marker dilution or obscuration by other serum proteins in the sample
Urine	Little evidence regarding the urinary proteome and its potential available in the literature
Saliva	Large amounts of salivary amylase, albumin, and immune-globulin contained in saliva that reduce the sensitivity
Pancreatic juice	Obtained through invasive procedures with associated risk of induced pancreatitis, contamination of the sample with duodenal and gastric juice, and reduced possibility of performing this technique in a large population of patients
Pancreatic cystic fluid	Limited by the amount of fluid aspirated and the size of the cyst, which can occasionally be insufficient

**Table 4:** Most promising serologic biomarkers and their advantages for clinical applications.

Author	Biomarkers	Advantages
**Papapanagiotou et al. (2018) [82]**	Osteonectin	Involved in the mechanisms of invasion and metastasis; detection of PC at early stage with good sensitivity (84.6%) and specificity (87.5%)
**Liu et al. (2016) [86]**	MUC16 and MUC5AC	MUC16 has a strong association with metastatic disease; MUC5AC has great efficacy in differentiating resectable early-stage PC from healthy controls (100% specificity in combination with CA 19-9)
**Honda et al. (2019) [88]**	APOE, APOA2, and APOC1	APOE and APOA2 have a high sensitivity and specificity in distinguishing PC from healthy controls; APOC1 correlated with poorer prognosis and independently predicted survival
**Yoneyama et al. (2016) [91]**	IGFBP-2 and IGFBP-3	High discriminatory power in distinguishing intraductal papillary mucinous neoplasm (IPMN) and controls
**Zhang et al. (2020) [93]**	Trefoil factors	Significant elevation in PC compared to chronic pancreatitis and benign controls; better accuracy when combined with CA 19-9 (AUC 0.93)
**He et al. (2011) [99]**	Osteopontin	Good ability to distinguish PC from chronic pancreatitis and healthy controls, both alone and in combination with CA 19-9
**Mitsunaga et al. (2010) [102]**	Laminin γ2 (LAMC2)	Inverse correlation with OS in patients with PC
**Jenkinson et al. (2016) [107]**	THBS1	Remarkable accuracy in the detection of PC (AUC 0.86)
**Nomura et al. (2008) [115]**	FGF-10/KGF-2	Significant levels in the sera of patients with PC compared to controls
**Yang et al. (2018) [119]**	Macrophage inhibitory cytokine-1/growth differentiation factor-1	Comparable diagnostic accuracy to CA 19-9 (sensitivity 80%, specificity 88%, diagnostic odds ratio 24.57) and moderately superior AUC (0.8945) in diagnosing PC; moderate predictive capacity to identify high-risk patients for developing cancer
**Torres et al. (2014) [120]**	CXCL11/interferon inducible T cell alpha chemokine	Prediction of treatment response to gemcitabine and erlotinib when overexpressed in PC
**Mehta et al. (2017) [130]**	Creatine, inosine, beta-sitosterol, sphinganine, glycocholic acid, and succinic acid	Great accuracy in distinguishing PC patients from healthy controls, diabetic patients, and colorectal cancer patients (AUC values of 0.997, 0.992, and 0.653, respectively)

**Table 5:** Other serologic biomarkers and their limitations for clinical applications.

Author	Biomarker	Disadvantages
**Chen et al. (2013) [95]**	Transthyretin	Expression also in endocrine tumors and epithelial ovarian carcinoma; heterogenous levels of expression in PC patients
**Joergensen et al. (2010) [98]**	TIMP-1	Lower sensitivity (47.1%), specificity (69.2%), and accuracy (AUC 0.64) than CA 19-9 in the detection of PC
**Gebauer et al. (2014) [103]**	CEACAM 5 and 6	Scant evidence for the detection of PC due to their overexpression in other solid organ malignancies
**Markocka-Maczka et al. (2003) [106]**	ICAM-1	Inability to distinguish between early- and late-stage PC
**Le Large et al. (2019) [108]**	THBS2	No difference in expression between PC and distal cholangiocarcinoma
**Hedström et al. (1996) [109]**	Trypsinogen-2, HSP27, serum amyloid A, and M2-pyruvate kinase	Elevation of their levels also in chronic pancreatitis and benign obstructive disease
**Yang et al. (2018) [119]**	TGF-β	Heterogeneous levels despite a significant correlation with poorer prognosis and reduced overall survival in PC patients compared to benign controls
**Miekus et al. (2021) [121]**	TNF- α	Extreme variability and lack of diagnostic ability for PC compared to biliary tract neoplasms and benign disease
**Sogawa et al. (2016) [124]**	C4b-binding protein a-chain and soluble gC1qR	A single study but there was significant elevation in PC compared to chronic pancreatitis or healthy controls
**Furukawa et al. (2015) [127]**	LRG1, sCD40L, and aminopeptidase N	Single small study, need for larger sample sizes and validation cohorts to confirm diagnostic efficacy

**Table 7:** Main evidence about advantages and disadvantages of exosome biomarkers in liquid biopsy.

Author	Biomarker	Sample	Advantages	Disadvantages
**Melo et al. (2015) [165]**, **Frampton et al. (2018) [166]**, **Lucien et al. (2019) [167]**	GPC-1	Serum exosomes	Significantly higher levels of GPC1+ exosomes in PC patients compared with benign pancreatic disease and healthy controls (*p* < 0.0001), with absolute sensitivity and positive and negative predictive value (100%)	Heterogenous results about the ability to distinguish PC from benign lesions using GPC1+ EVs
**Flammang et al. (2020) [173]**	miR-192-5p	Serum exosomes	Diagnostic accuracy comparable to CA 19-9 in distinguishing PC patients from healthy controls (AUC = 0.83, *p* = 0.0004)	Not able to distinguish patients with PC and chronic pancreatitis
**Reese et al. (2020) [174]**	miR-200b and miR-200c	Serum exosomes	Overexpression in PC patients compared with healthy controls (*p* < 0.001; *p* = 0.024) and chronic pancreatitis (*p* = 0.005; *p* = 0.19) with a combined diagnostic accuracy along with CA 19-9 of 97% (*p* < 0.0001) in predicting PC	Circulating exosomal miR-200c (AUC = 0.70) did not reach the previously reported diagnostic accuracy of miR-200c derived from tissue (AUC = 0.84) or blood serum (AUC = 0.78) in differentiating between PC and non-PC
**Pu et al. (2020) [176]**	miR-21 and miR-10b miR-3940-5p, miR-8069	Serum exosomes	Improved diagnostic value (AUC = 0.791, *p* < 0.0001) in differentiating early-stage PC from healthy controls and advanced-stage PC	Future studies should examine the levels of these miRs before and after treatment, such as surgery, radiotherapy, chemotherapy, and molecular targeted therapy
**Yoshizawa et al. (2020) [179]**	miR-3940-5p/miR-8069 ratio	Urine exosomes	Improved sensitivity (93.0%) and positive predictive value (100%) in combination with elevated CA 19-9 at a relatively early stage of disease	Small number of samples; lack of data about its relationship with PC prognosis or possible changes after therapy
**Ankeny et al. (2016) [181]**	KRAS mutations	Circulating tumor cells	Good diagnostic tool for PC (sensitivity = 75.0%, specificity = 96.4%, AUROC = 0.867, *p* < 0.001) with a cut-off of ≥3 CTCs in 4 mL of venous blood to discriminate between local/regional and metastatic disease (AUROC = 0.885 and *p* < 0.001)	Use of an epithelial surface marker (EpCAM) for CTC capture that potentially led to decreased sensitivity secondary to loss of CTCs expressing non-epithelial surface markers
**Terasawa et al. (2019) [183]**	KRAS mutations	Urinary cell-free DNA	Potential role of urinary liquid biopsy in PC with detection rate and sensitivity comparable to plasma liquid biopsy	Heterogeneous results according to renal functions, single center study, small number of patients enrolled, patient characteristics also biased, no early disease stage

**Table 8:** Main evidence regarding non-coding RNAs.

Author	Biomarker	Sample	Results
**Wang et al. (2009) [198]**	miR-21, miR-210, miR-155, and miR-196a	Serum	Sensitivity of 64% and specificity of 89% in the identification of early PC
**Abue et al. (2015) [199]**	miR-21	Serum	Role in distinguishing PC patients from healthy controls (*p* < 0.01); higher levels at advanced stage of disease (*p* < 0.05), metastasis to lymph node and liver (*p* < 0.01), and shorter survival (*p* < 0.01) in patients with PC
**Guz et al. (2021) [200]** **Ho et al. (2010) [201]**	miR-210-3p	Serum	Increased levels in PC compared with chronic pancreatitis patients (*p* = 0.015) and control group (*p* < 0.001); positive correlation with alkaline phosphatase (r = 0.605; *p* = 0.022) and γ-glutamyltranspeptidase (r = 0.529; *p* = 0.029)
**Guo et al. (2018) [206]** **Shuai et al. (2020) [207]**	lncRNA SNHG15	Serum	Upregulation in differentiating PC from healthy controls; role in predicting tumor differentiation (*p* = 0.000), lymph node metastasis (*p* = 0.001), tumor stage (*p* = 0.005), and shorter overall survival (*p* = 0.003)
**Debernardi et al. (2015) [213]**	miR-143, miR-223, and miR-30e	Urine	Overexpression in stage I PC compared with healthy controls (*p* = 0.022, 0.035, and 0.04, respectively) and with stages II-IV PC (*p* = 0.025, 0.013, and 0.008, respectively)
**Sadakari et al. (2010) [214]**	miRNA-21 and miRNA-155	Pancreatic juice	Significantly higher in PC than chronic pancreatitis (*p* < 0.001 and *p* = 0.008, respectively); AUC of 0.90 and 0.89 and accuracy of 83% and 89%, respectively
**Wang et al. (2014) [215]**	miR-205, miR-210, miR-492, and miR-1427	Pancreatic juice	Prediction of PC with specificity of 88% and sensitivity of 87% when combined; higher levels of expression of miR-205 and miR-210 predicted lymph node metastasis
**Humeau et al. (2015) [216]**	miR-21, miR-23a, miR-23b and miR-29c	Saliva	Upregulation in unresectable PC compared to control with sensitivities of 71.4%, 85.7%, 85,7%, and 57% and specificity of 100%
**Xie et al. (2015) [217]**	miR-3679-5p and miR-940	Saliva	Role in distinguishing resectable pancreatic cancer within the three categories (PC, benign pancreatic tumors, healthy controls) with sensitivities of 72.5%, 62.5%, and 70.0% and specificities of 70.0%, 80.0%, and 70.0%, respectively
**Matthaei et al. (2012) [218]**	miR-18a, miR-24, miR-30a-3p, miR-92a, miR-99b, miR-106b, miR-142-3p, miR-342-3p, miR-532-3p	Cystic fluid	Role in the prediction of cyst pathology implying resection vs. conservative management with a sensitivity of 89%, a specificity of 100%, and area under the curve of 1

## Data Availability

No new data were created or analyzed in this study. Data sharing is not applicable to this article.
